# ErtlFunctionalGroupsFinder: automated rule-based functional group detection with the Chemistry Development Kit (CDK)

**DOI:** 10.1186/s13321-019-0361-8

**Published:** 2019-06-04

**Authors:** Sebastian Fritsch, Stefan Neumann, Jonas Schaub, Christoph Steinbeck, Achim Zielesny

**Affiliations:** 1 GNWI – Gesellschaft für naturwissenschaftliche Informatik, Oer-Erkenschwick, Germany; 2 Institute for Bioinformatics and Chemoinformatics, Westphalian University of Applied Sciences, August-Schmidt-Ring 10, 45665 Recklinghausen, Germany; 3 Institute for Inorganic and Analytical Chemistry, Friedrich-Schiller-University, Jena, Germany

**Keywords:** Chemistry Development Kit, CDK, Functional group, Aromaticity, Electron donation, Cycle finder

## Abstract

The Ertl algorithm for automated functional groups (FG) detection and extraction of organic molecules is implemented on the basis of the Chemistry Development Kit (CDK). A distinct impact of the chosen CDK aromaticity model is demonstrated by an FG analysis of the ChEMBL database compounds. The average performance of less than a millisecond for a single-molecule FG extraction allows for fast processing of even large compound databases.

## Introduction

Functional groups (abbreviated FG) are an important concept of organic chemistry. They allow for a systematic and (in many cases) adequate molecular categorization according to a molecule’s reactivity and its chemical properties. Moreover, the FG concept may be successfully exploited across a wide range of molecular research, e.g. to construct quantitative structure–activity relationships (QSAR) in order to support drug discovery. In a recent publication [1] Peter Ertl proposed a new purely rule-driven approach to identify FGs of an organic molecule. This effort may be regarded as the first genuine algorithmic method to tackle FG identification in contrast to the common manual FG definition performed by chemists. A first open implementation of the Ertl algorithm (denoted *IFG*—*Identify Functional Groups*) was realized by Guillaume Godin and Richard Hall for the RDKit package [2].

In this work, the Ertl algorithm for automated FG detection and extraction is implemented on the basis of the Chemistry Development Kit (CDK) [3–6] with a new Java class *ErtlFunctionalGroupsFinder* to extend its open applicability for molecular research. The concrete CDK implementation and the distinct impact of the chosen CDK aromaticity model on FG detection as well as a comparison with the *IFG* RDKit implementation are discussed in detail. Due to the average performance of less than a millisecond for a single-molecule FG extraction using a single standard workstation processor core, *ErtlFunctionalGroupsFinder* may be used to process even large databases with a tenth of millions of molecules within an hour.

## Implementation details

The implementation of the Ertl algorithm is divided into three consecutive steps. Step I marks all atoms within a molecule that meet the Ertl rules. Step II detects groups of connected marked atoms and extracts each group as a FG including information about its environment. The final step III applies the Ertl generalization scheme to the detected FGs.

## Step I

*AtomContainer* is the basic molecule representation class of the CDK. In order to mark atoms according to the Ertl rules, atomic connectivity information of an *AtomContainer* has to be queried (e.g. ‘Is atom A connected to atom B?’). Since *AtomContainer* internally uses edge-lists the marking procedure would scale linearly with the number of a molecule’s bonds. To avoid this inefficiency, the CDK utility class *GraphUtil* is alternatively invoked to generate an adjacency list with a complementing edge-to-bond map for quick atomic connectivity access. The marking procedure iterates over all non-aromatic atoms of the molecule in the order given by its *AtomContainer*. Heteroatoms (unlike carbon or hydrogen) are identified by their atomic number, whereas for carbons all neighboring atoms and bonds are evaluated in a successive manner according to the Ertl rules. For special treatment in the following steps, aromatic heteroatoms are collected in a separate manner and marked carbons in carbonyl groups are specifically labeled. If not already the case, explicit hydrogens are set as an implicit property of the connected parent atoms. The iteration result is a set of marked atoms which acts as a basis for the following FG extraction in Step II.

## Step II

To identify groups of connected marked atoms in combination with their merger into FGs, a single unprocessed marked atom is picked as a starting point for a new FG. Then an iterative breadth-first search (BFS) based on the adjacency list explores all neighboring atoms and expands the group by adding connected marked atoms until unmarked carbons or aromatic heteroatoms are reached which form the FG’s environment. These terminal atoms are not included in the FG themselves but their aromaticity and bonding information is extracted and attributed to their connected marked atoms. The extraction process keeps the aromaticity assignments and molecular orbitals of the molecule under investigation. In addition, aromatic heteroatoms that are not included in a group are extracted separately as single-atom FGs. Once all marked atoms are processed, the complete list of FGs (including environmental carbon information) of the molecule is obtained in form of a list of *AtomContainers*.

## Step III

The final generalization step processes all extracted FGs separately according to the Ertl generalization scheme. Each FG is represented by an *AtomContainer* that contains marked atoms, their connecting bonds, and information on (former) neighboring environmental carbons. The information on environmental carbons comprises their location and their aromaticity as derived from the molecule under investigation. First, all exceptional cases are addressed where the FG contains a single marked atom only. This includes single-atomic nitrogen or oxygen FGs with one environmental carbon, simple thiols, and secondary amines or single aromatic hetero-atom FGs. Then an iteration over all atoms in the FG is performed. In case of a heteroatom, all hydrogens are replaced by new R-atoms which are implemented as instances of the *PseudoAtom* class—while oxygens in hydroxyl groups retain their hydrogens as an exception. According to the generalization scheme, any environmental information about carbon atoms is deleted with the exception of previously-labeled carbons in carbonyls which are replaced by R-atoms. The resulting generalized FGs of the molecule are finally returned as an *AtomContainer* list.

*ErtlFunctionalGroupsFinder* provides two basic processing modes which can be defined via the class constructor: The default *generalization* mode generalizes all detected FGs as outlined above whereas the *no*-*generalization* mode replaces generalization with an alternative outline of the environmental information with distinct atoms and bonds including their original aromaticity.

## Aromaticity models

**Fig. 1:**
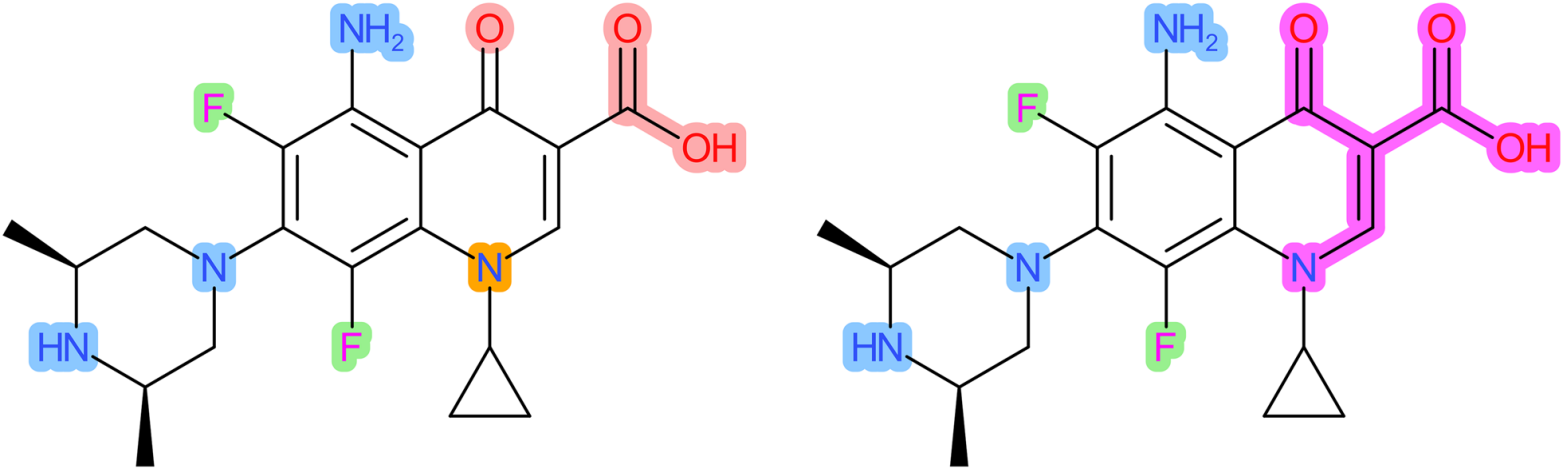
Influence of the different CDK electron donation types on FG detection (identified FGs are being highlighted by a colored background, the same cycle finder *Cycles*.*all* algorithm is used for all electron donation types). Left: The *daylight* electron donation type assigns a fully aromatic ring structure with corresponding FGs. Right: The electron donation types *cdk, cdkAllowingExocyclic* and *piBonds* assign an aromatic benzene ring plus an annulated aliphatic ring with only one resulting larger FG on the right (highlighted in pink) instead of three corresponding FGs (highlighted in red and orange) for the *daylight* electron donation

For application of *ErtlFunctionalGroupsFinder*, a molecule has to be provided as an *AtomContainer* instance with well-defined CDK atom types and a distinct aromaticity model (electron donation type plus cycle finder algorithm for ring detection)—note that the *AtomContainer* instance is not allowed to contain any charged, metal or metalloid atoms or multiple unconnected structures as described in the preprocessing steps outlined in [1]. The CDK provides four electron donation types: (1) *ElectronDonation.cdk*—CDK’s model which derives each atom’s electron contribution from the CDK atom types, (2) *ElectronDonation.cdkAllowingExocyclic*—as aforementioned but allows electron contributions from exocyclic pi-bonds, (3) *ElectronDonation.piBonds*—a simple model that only allows electron contributions from cyclic pi-bonds, and (4) *ElectronDonation.daylight*—a model that closely follows the one used by Daylight in the generation of SMILES. For ring detection, various cycle finder algorithms are available: *Cycles.all*, *Cycles.mcb*, *Cycles.relevant*, *Cycles.essential*, *Cycles.tripleShort*, *Cycles.vertexShort* and *Cycles.edgeShort*. The aromaticity model may distinctly influence FG detection since the marking procedure of step I depends on the separation of aromatic and aliphatic bonds/atoms as outlined above (see Fig. 1).

## Results and discussion

**Fig. 2:**
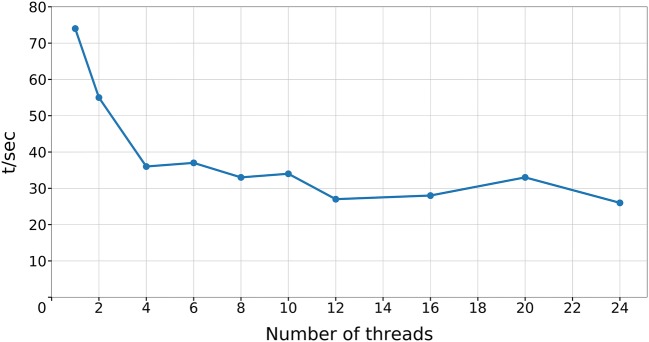
Performance snapshot of *ErtlFunctionalGroupsFinder* for FG extraction from 1.8 million ChEMBL compounds in dependence of the number of parallelized processing threads

The CDK implementation of the Ertl algorithm allows for a fast FG extraction. A performance snapshot with the *ErtlFunctionalGroupsFinderPerformanceTest* tool exhibits an in-memory processing speed of 74 s for 1.8 million ChEMBL compounds [7, 8] using a single core of an Intel Xeon E5-2697 v2 workstation CPU [9]. This corresponds to a single-core processing speed of more than 50 million molecules per hour. The FG extraction performance of parallelized *ErtlFunctionalGroupsFinder* threads with equal shares of the ChEMBL molecules (using the same hardware) is shown in Fig. 2: an initial distinct decrease of processing time flattens to only minor performance enhancements beyond four parallelized threads—the efficient FG extraction with four parallelized threads allows to process more than 150 million molecules per hour.

**Fig. 3:**
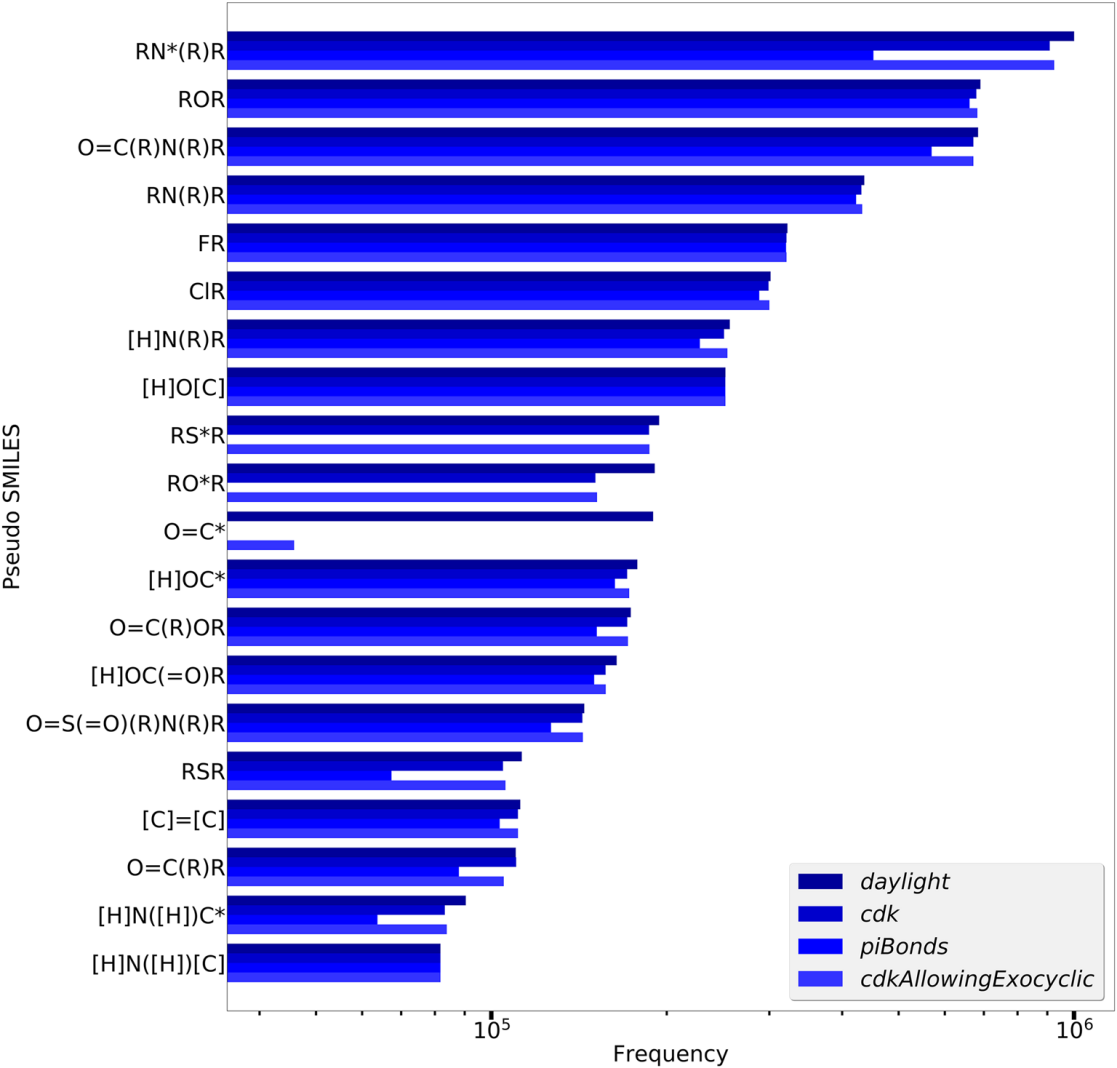
Frequencies of the twenty most frequent FGs of 1.8 million ChEMBL compounds for different electron donation types with cycle finder *Cycles.all*

The *ErtlFunctionalGroupsFinderEvaluationTest* tool generates FG extraction results where FGs are represented as pseudo SMILES strings with aromatic atoms marked by an asterisk and pseudo-atoms indicated by character R. In a preliminary step *ErtlFunctionalGroupsFinderEvaluationTest* excludes molecules with metal or metalloid atoms, selects the largest part from compounds with multiple unconnected structures and neutralizes charged atoms. The latter is performed by zeroing the formal atomic charges and filling up free valences with hydrogen atoms (according to the CDK atom types). This procedure allows a more general charge treatment than a pre-defined transformation list but may produce “wrong” structures, e.g. it turns a nitro NO_2_ group into pseudo SMILES “[H]O[N](= O)R” with an uncharged four-bonded nitrogen atom (other examples are “R[N](R)(R)R”, “[C]#[N]R” or “RS(R)(R)R”). Thus an improved charge neutralization scheme is desirable for future implementations. Figure 3 shows the twenty most frequently detected FGs of 1.8 million ChEMBL compounds for the *daylight* electron donation in comparison to the findings with the three other electron donation types (cycle finder *Cycles.all* algorithm is used which is substituted by *Cycles.vertexShort* in case of a CDK intractable exception).

The most common FG for *daylight* electron donation is a tertiary amine with an aromatic central nitrogen atom, followed by an ether group, an amide group and a tertiary amine with a non-aromatic central atom. Differences between the four electron donation types are obvious with some striking examples: The “O=C*” FG (representing a carbonyl group containing an aromatic carbon atom) is frequent for the *daylight* and *cdkAllowingExocyclic* type but does not appear at all for *cdk* and *piBonds.* The *cdk* type does not allow atoms with exocyclic double or triple bonds in aromatic systems. Therefore, a carbon atom connected to carbonyl oxygen is not considered aromatic in any case. For *piBonds* all possibly aromatic atoms must be connected to a cyclic pi-bond which is impossible for the carbon atom in a carbonyl group (and also for oxygen and sulfur atoms, compare FGs “RO*R” and “RS*R” in Fig. 3). Type *cdkAllowingExocyclic*, on the other hand, allows electron contributions from exocyclic pi-bonds and the *daylight* type tolerates a carbonyl carbon in an aromatic system but considers its electron contribution to be zero since the oxygen atom is more electronegative. The detected most frequent ChEMBL FGs are in correspondence with the findings in [1] obtained from a specific bioactive subset of ChEMBL. The most frequent “RN*(R)R” aromatic amine FG is not explicitly mentioned in [1] but published in the supplementary file.

**Fig. 4:**
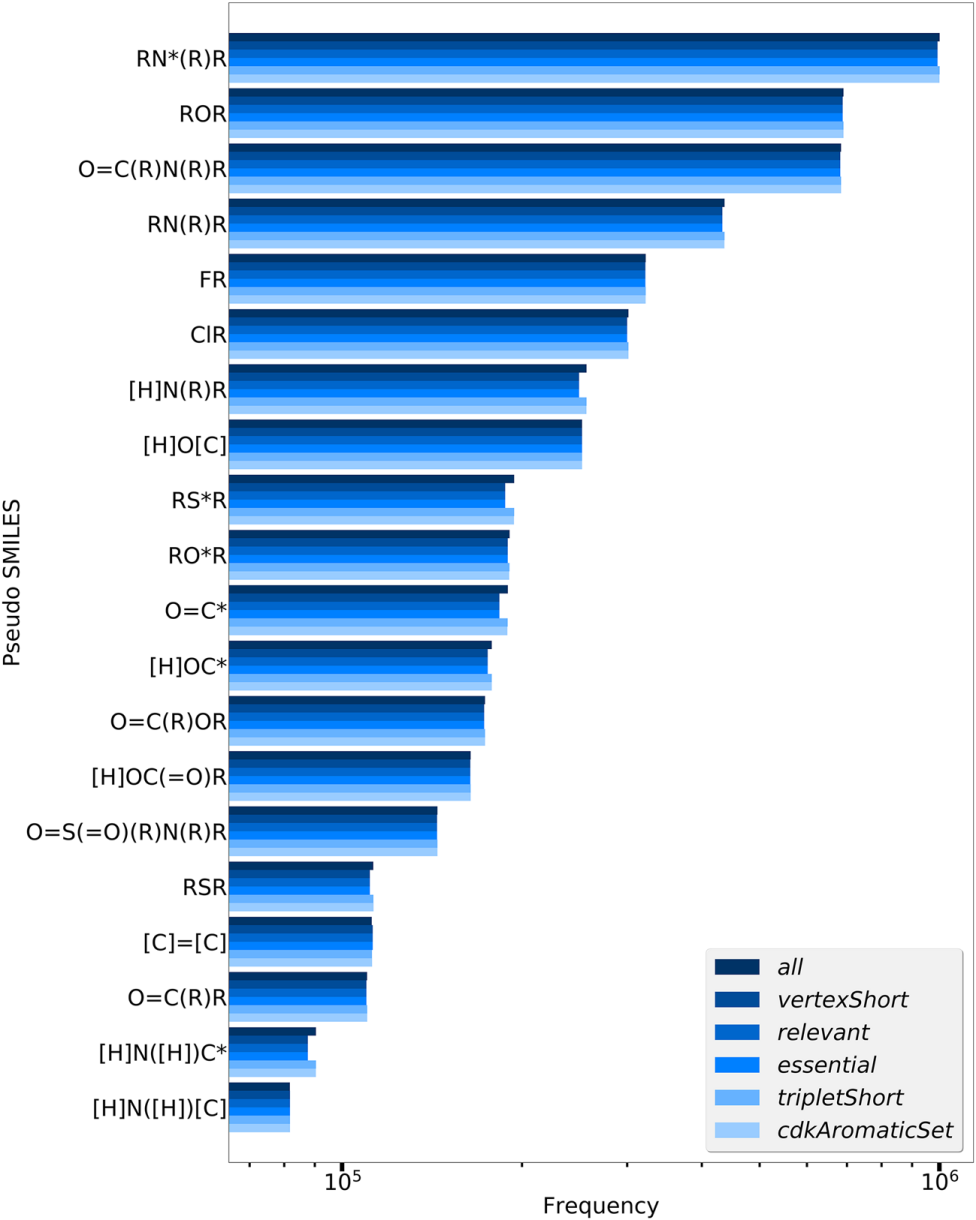
Frequencies of the twenty most frequent FGs of 1.8 million ChEMBL compounds for different cycle finder algorithms and the *daylight* electron donation model

Figure 4 depicts the cycle finder algorithm influence on the resulting FG frequencies for the *daylight* electron donation type with a subset of the available CDK cycle finder algorithms. Compared to the differences between the electron donation types, the cycle finder algorithm influence is of minor importance but nonetheless leads to deviations of about 4% in FG frequencies (e.g. the frequency of ‘RS*R’ varies between 187,503 and 194,136 molecules containing this functional group).

**Fig. 5:**
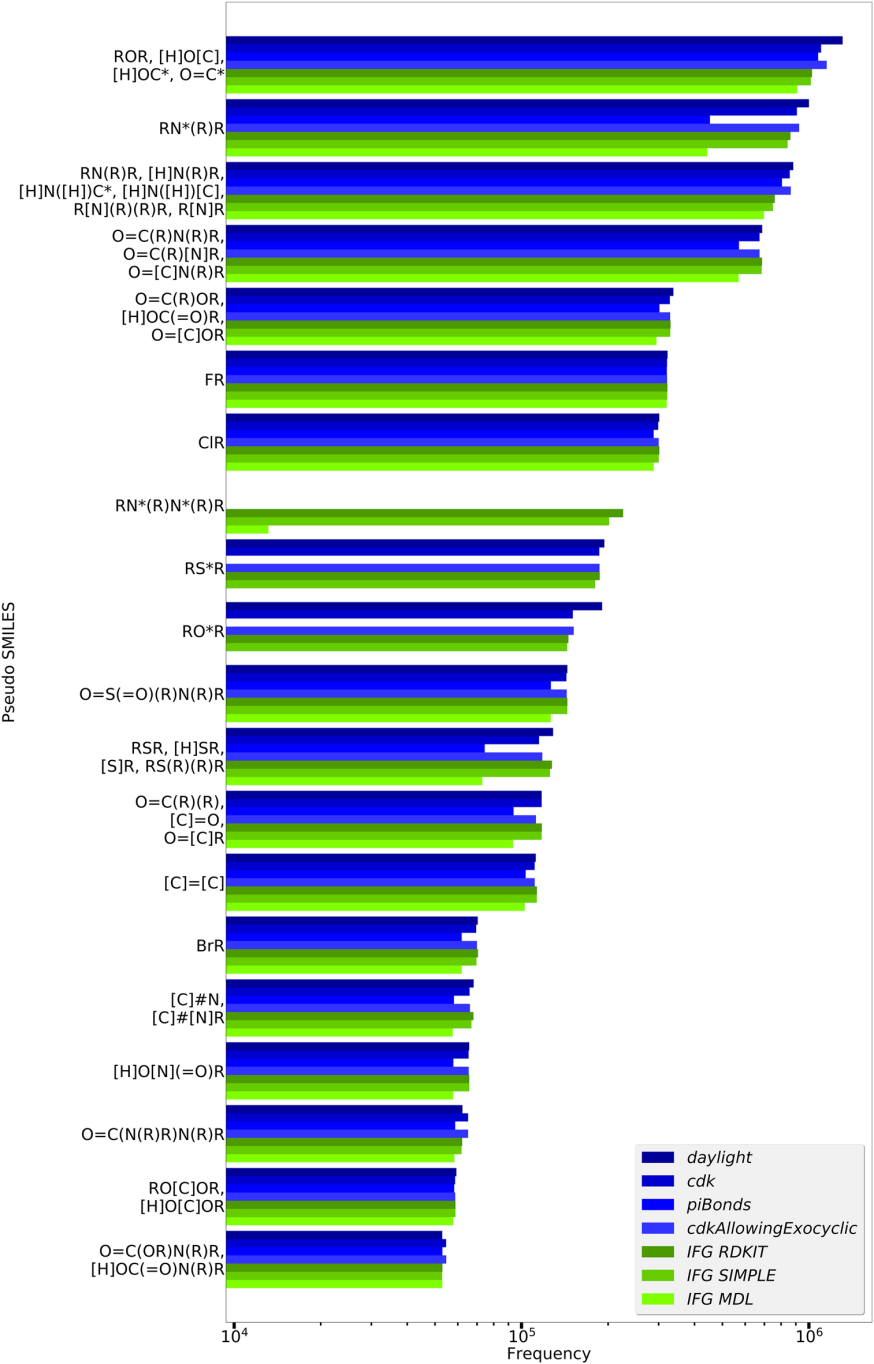
Comparison of FG detection between *ErtlFunctionalGroupsFinder* (blue bars) and *IFG* RDKit (green bars) for different aromaticity/electron donation models (the RDKit aromaticity model labels are abbreviated like *IFG AROMATICITY_RDKIT* to simply *IFG RDKIT*). For details see text

We originally intended this article to purely describe our CDK implementation of the Ertl algorithm. During the publication process, one of the reviewers requested a comparison with the open *IFG* RDKit implementation, not in terms of the execution time but in terms of providing similar results. We agreed to include the following evaluation of both implementations, but would like to highlight some caveats. A direct one-to-one FG detection comparison of *ErtlFunctionalGroupsFinder* with the *IFG* RDKit implementation suffers from the fact that *IFG* RDKit does not provide generalized FGs according to the Ertl generalization scheme. But the resulting *IFG* RDKit SMILES string with only marked atoms can be regarded as an approximated generalized FG representation (since it does not contain environmental information) thus it can be mapped to a set of multiple pseudo SMILES FGs generated by *ErtlFunctionalGroupsFinderEvaluationTest*. As an example the *IFG* RDKit SMILES string “O=CO” represents a carboxyl group, an ester group and a formic acid ester group which correspond to pseudo SMILES FGs “[H]OC(=O)R”, “O=C(R)OR” and “O=[C]OR” of *ErtlFunctionalGroupsFinderEvaluationTest*. Figure 5 shows the comparison for the twenty most frequent FGs detected in 1.8 million ChEMBL compounds using *IFG* RDKit with the AROMATICITY_RDKIT aromaticity model (plus standard RDKit valence model and standard cycle finder algorithm): Each *IFG* RDKit FG is represented by the corresponding set of *ErtlFunctionalGroupsFinderEvaluationTest* pseudo SMILES FGs with their individual frequencies summed up.

The FG detection results of both implementations are in good general agreement: The most common FG detected by *IFG* RDKit is “O” (representing a single oxygen atom with unknown connections). It equals the generalized *ErtlFunctionalGroupsFinderEvaluationTest* FGs representing an ether group (pseudo SMILES “ROR”), hydroxyl groups connected to aromatic carbon atoms (pseudo SMILES “[H]OC*”) or aliphatic carbon atoms (pseudo SMILES “[H]O[C]”) and a carbonyl group containing an aromatic carbon atom (pseudo SMILES “O = C*”). A striking deviation is the chemically meaningful FG “RN*(R)N*(R)R” which represents two bonded aromatic nitrogen atoms (e.g. found in pyridazine): While this FG is frequently detected with *IFG* RDKit it is not at all found by *ErtlFunctionalGroupsFinder*—but this detection failure of the latter is in concordance with the Ertl algorithm which defines that aromatic heteroatoms are to be collected as single atoms if no aliphatic group is connected. Last but not least the total numbers of different identified FGs are smaller for *IFG* RDKit (11.000 with *AROMATICITY_RDKIT* up to 43.000 with *AROMATICITY_MDL*) compared to *ErtlFunctionalGroupsFinder* (41.000 with *daylight* up to 134.000 with *piBonds*) which can be traced to the different FG output representations.

As a résumé, chemical FG detection remains challenging—and different open implementations of the Ertl algorithm are a true virtue, not only for general comparisons but especially for their different oddities and subtleties. For example, the *ErtlFunctionalGroupsFinderEvaluationTest* pseudo SMILES FG “R[N]R” is not straight forward to comprehend, the same applies to “[S]R”, “O=C(R)[N]R” or “O=[C]R” (the latter does not represent an aldehyde group since this FG is represented by “[C]=O” thus it may be an artefact due to a SD file error or originate from an amide group containing an aromatic nitrogen atom). Further investigations of these rare problems may lead to an improved structural pre-processing as well as possible useful extensions of the Ertl FG detection rules.

The numerous possible applications of FG detection in molecular research are widespread: FGs may be regarded as “intrinsic seeds” for proper fragmentation of molecules. The FG sets of single molecules up to large molecule collections can be regarded as chemically meaningful “feature vectors” or “fingerprints”: This qualifies their use for molecular comparison, clustering/classification or ranking purposes (e.g. based on “overlap in FG space”) and may substantially improve QSAR/QSPR research, especially the growing and increasingly complex machine learning approaches.

The *ErtlFunctionalGroupsFinder* LGPL Java class code is openly available from its project page. It is recommended to place this java class in the *tools* package of the *cdk*-*extra* module. *ErtlFunctionalGroupsFinder* depends only on CDK base classes and interfaces as well as *GraphUtil* for quick connectivity queries. Unit tests for 20 compounds given in [1] (with the *daylight* electron donation model and cycle finder *Cylces.all* for aromaticity detection) are implemented: They demonstrate adequate FG extraction of the new implementation by comparison of the expected FGs according to the Ertl rules with the actual results. The open tools *ErtlFunctionalGroupsFinderPerformanceTest* and *ErtlFunctionalGroupsFinderEvaluationTest* provide detailed sample code for using the new functionality. An integration into future CDK releases is requested and will hopefully be approved by the CDK community.

## Data Availability

Project name: ErtlFunctionalGroupsFinder. Project home page: https://github.com/zielesny/ErtlFunctionalGroupsFinder. Operating system(s): Platform independent. Programming language: Java. Other requirements: None. License: LGPL.
